# Competitive Carboxylate–Silicate Binding at Iron Oxyhydroxide Surfaces

**DOI:** 10.1021/acs.langmuir.1c02261

**Published:** 2021-10-29

**Authors:** Wei Cheng, Rémi Marsac, Khalil Hanna, Jean-François Boily

**Affiliations:** † College of Resources and Environmental Science, South-Central University for Nationalities, Wuhan 430074, P.R. China; ‡ Université Rennes, CNRS, Géosciences Rennes−UMR 6118, Rennes F-35000, France; § Université Rennes, Ecole Nationale Supérieure de Chimie de Rennes, UMR CNRS 6226, 11 Allée de Beaulieu, Rennes Cedex 7 F-35708, France; ∥ Institut Universitaire de France (IUF), MESRI, 1 rue Descartes, Paris 75231, France; ⊥ Department of Chemistry, Umeå University, Umeå SE-901 87, Sweden

## Abstract

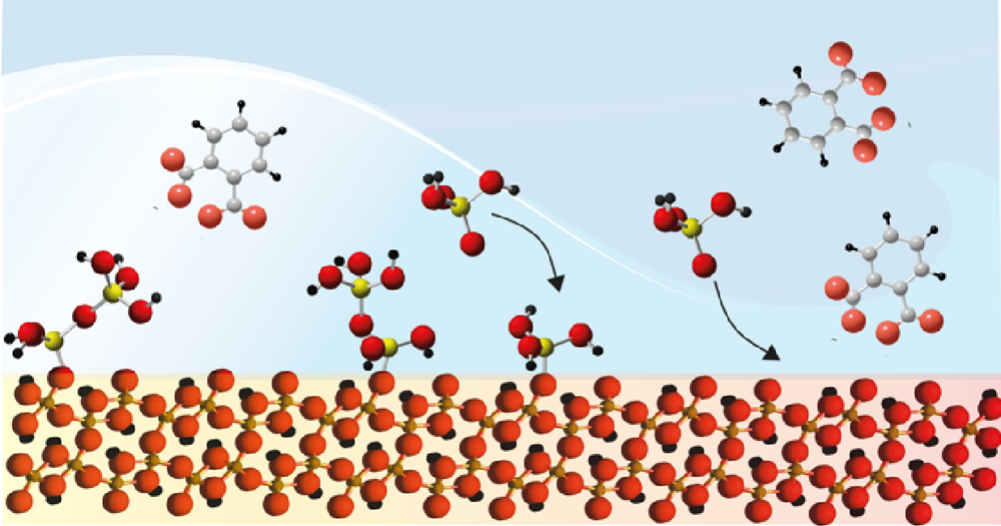

Dissolved silicate ions in wet and dry soils can determine the fate of organic contaminants via competitive binding. While fundamental surface science studies have advanced knowledge of binding in competitive systems, little is still known about the ranges of solution conditions, the time dependence, and the molecular processes controlling competitive silicate–organic binding on minerals. Here we address these issues by describing the competitive adsorption of dissolved silicate and of phthalic acid (PA), a model carboxylate-bearing organic contaminant, onto goethite, a representative natural iron oxyhydroxide nanomineral. Using surface complexation thermodynamic modeling of batch adsorption data and chemometric analyses of vibrational spectra, we find that silicate concentrations representative of natural waters (50–1000 μM) can displace PA bound at goethite surfaces. Below pH ∼8, where PA binds, every bound Si atom removes ∼0.3 PA molecule by competing with reactive singly coordinated hydroxo groups (−OH) on goethite. Long-term (30 days) reaction time and a high silicate concentration (1000 μM) favored silicate polymer formation, and increased silicate while decreasing PA loadings. The multisite complexation model predicted PA and silicate binding in terms of the competition for −OH groups without involving PA/silicate interactions, and in terms of a lowering of outer-Helmholtz potentials of the goethite surface by these anions. The model predicted that silicate binding lowered loadings of PA species, and whose two carboxylate groups are hydrogen- (HB) and metal-bonded (MB) with goethite. Vibrational spectra of dried samples revealed that the loss of water favored greater proportions of MB over HB species, and these coexisted with predominantly monomeric silicate species. These findings underscored the need to develop models for a wider range of organic contaminants in soils exposed to silicate species and undergoing wet–dry cycles.

## 1 Introduction

Silicate is one of the most widely distributed major oxyanion in soils and natural waters.^1^ As a weathering product of silicate rocks, it is typically present at sub- to millimolar concentrations (*e.g*., 0.17–1.24 mM).^2^ Silicate ions strongly adsorbed to mineral surfaces (*e.g.,* iron oxyhydroxides, clays) can block reactive centers and thereby alter contaminant and element cycling and transport in nature.^3−7^ Although other naturally occurring anions (*e.g.,* sulfate, phosphate) also have the ability to block reaction centers, silicate stands out for its ability to form polymeric coatings and for being an ion of widespread occurrence in natural waters. As such, understanding how silicates alter mineral surface site reactivity,^3,8^ including associated mineralogical transformations,^9−11^ is key to improving mass transport predictions in terrestrial and aquatic environments. Such predictions deserve a special focus on nanosized iron (oxyhydr)oxides given the reactivity and widespread occurrence of these particles in nature.

**Figure 1:**
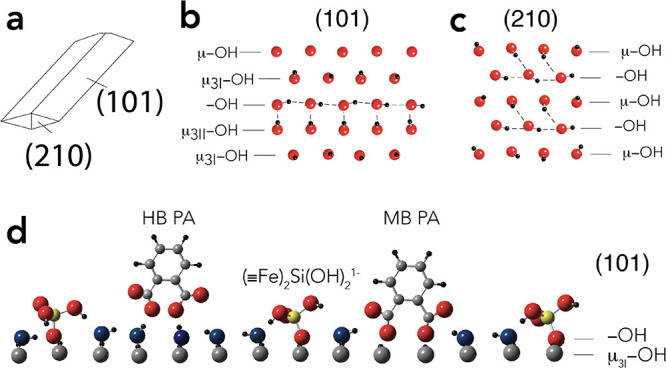
Schematic representation of (a) crystal habit and (b–d) disposition of species and hydrogen bonding populations on dominant crystallographic faces of goethite. (a) Idealized crystal habit showing dominant (101) and (210) faces. (b, c) Disposition hydroxo groups on the (b) (101) and (c) (210) faces. See Song and Boily^41^ for details. (d) Disposition of monomeric silicate species and PA along a row of −OH groups on the (101) face.

Silicate anions form strong metal-bonded (MB) species on minerals over a very broad range of pH values, a result of ligand exchange reactions involving surface hydroxo (OH) groups (Figure 1).^8,12−14^ Species formed on a variety of iron (oxyhydr)oxide minerals (*e.g*., iron hydroxide,^15^ goethite,^7,8,12,13,16^ hematite,^4,5,12^ magnetite,^3,6,12^ and ferrihydrite^9,11,17−19^) have previously been studied using vibrational spectroscopy^13,18,20,19^ and molecular simulations.^8,17,18^ Moreover, the occurrence of these species can be predicted using surface complexation modeling.^8,12,15−17^ A model that we recently developed^21^ accounts for the pH and loading dependence of monomeric silicate species and their transformation to polymeric species. Considerably less is, however, known about (i) how silicate species impact the fate of other compounds—especially organic contaminants—competing for the same mineral surface sites, (ii) whether polymeric silicate species^5,13,20^ appearing over the course of a long reaction time (days to weeks) affect this competition, and (iii) how the loss of interfacial waters through (episodic or cyclic) drying, which is typical of terrestrial environments (*e.g.,* vadose zone of soils), affects the interfacial speciation. Understanding these competitive binding reactions is central to predicting the fate of compounds in terrestrial environments, where mineral surfaces are commonly exposed to silicate species.

Some of the better known competitive systems involve mixed oxyanions such as arsenic^5,7,18,22^ and selenium^4,6^ species. The literature reveals, for example, that monomeric surface silicate species effectively decrease arsenite^7,18,22^ and selenite^4^ loadings but negligibly affect arsenate loadings.^18^ Other studies that also have evaluated the effects of silicate polymerization on competitive binding reported contrasting findings. For example, Christl *et al.*(5) reported that silicate polymers on hematite lower arsenite and arsenate binding, but Luxton *et al.*(7) also reported that polymers do not significantly alter arsenite binding rates and loadings compared to monomers. Swedlund^23^ found, on the other hand, that monomeric silicate surface species inhibit arsenic adsorption to a greater extent than polymers. Of note, few studies used surface complexation models to quantify these competitive binding effects. Those that have^4,6,23^ do not account for silicate polymers.

Along the same vein, there is a growing need to understand how silicates impact of fate of organic contaminants in nature, especially contaminants with the environmentally important carboxylate functional groups that target the same reactive OH binding sites as oxo functional groups of dissolved silicate species (Figure 1). For example, Rusch et al.^24^ found that salicylate binding to goethite-coated sand remobilized adsorbed silicate, and Roonasi et al.^25^ found that silicate had only a minor impact on magnetite-bound oleate but effectively reduced oleate loadings when magnetite was initially preequilibrated with silicate. Despite these types of efforts, little is still known about the range of solution conditions, the time dependence, and the molecular processes controlling these competitive reactions. To achieve this, knowledge of the coordination modes of silicate and carboxylate-bearing organic species is needed. This can be achieved by identifying ligands forming (i) metal-bonded (MB; inner-sphere (IS)), (ii) hydrogen-bonded (HB; direct H-bond to surface (hydr)oxo group), or (iii) bound outer-sphere (OS; separated by at least one hydration sheath) complexes. Of note, the relative importance of these species is affected by pH (MB at low and HB/OS at high pH), ionic strength (MB at high and HB/OS at low ionic strength), as well as the interplay of mineral surface and ligand structures. Fundamental surface science studies on low molecular weight carboxylic acids, which are the focus of this work, have been particularly beneficial along this front.^26−34^

Here, we address three leading issues—competitive binding, aging, and drying—by examining the competitive adsorption of silicate and a model carboxylic acid on goethite. We chose phthalic acid (PA; benzene-1,2-dicarboxylic acid) because it is a typical carboxylate- and phenyl ring-bearing soluble organic contaminant in soils and groundwater. PA has a strong capability for forming MB complexes with surface metal ions (e.g., Fe^3+^ on iron oxyhydroxides), and of forming HB/OS surface complexes on minerals.^34^ Moreover, PA is an endocrine-disrupting agent^35^ that can originate from plastic debris, so its detection in soils, freshwater, and seawater^36,37^ raises grave environmental concerns.^38,39^ Goethite (α-FeOOH) was, in turn, chosen as a representative iron oxyhydroxide for soils and sediments.^40^ Knowledge of dominant known crystal faces of synthetic goethite nanoparticles also facilitates the interpretation of plausible silicate and PA species, notably those disposed along rows of regularly spaced reaction centers on dominant crystallographic faces (Figure 1).

In this study, pH- and concentration-dependent silicate and PA loadings were investigated for 1 and 30 day reaction periods. This strategy allowed us to account for competitive effects resulting from monomeric silicate species formed at short reaction times compared to those exerted by polymeric silicate species, which appear over longer reaction times. Thermodynamic modeling of batch adsorption data and chemometric analyses and interpretations of vibrational spectra identified dominant molecular species of silicate and PA formed under wet conditions typical of water-saturated soils. Additionally, the spectra of dry goethite samples revealed how dehydration altered the molecular scale speciation of coexisting PA and silicate species. These observations are not only directly relevant to soils undergoing wet–dry cycling but also add further insight into the ever-growing literature^42,26,43−49,27^ on molecular-scale phenomena driving carboxylate-bearing organic ligand adsorption to mineral surfaces.

## 2 Materials and Methods

### 2.1 Materials

All reagents were purchased from Sigma-Aldrich and used without further purification. All solutions were prepared with ultrapure water. The stock solution of silicate (2 mM) was made from Na_2_SiO_3_·9H_2_O, and the stock solution of PA (1 mM) was made from phthalic acid. Fourier transform infrared (FTIR) measurements in the Si–O stretching region did not reveal any polymeric species in these stock solutions. This aligns with the solution work^50^ that shows that polymeric species form in solutions of larger silicate concentrations, as well as with our previous work^13^ that shows that polymeric species on goethite only appeared in solutions exceeding 2 mM silicate.

### 2.2 Goethite Synthesis and Characterization

Goethite was synthesized as in previous studies.^28,51^ Briefly, 400 mL of a 2.5 M sodium hydroxide solution was titrated to 500 mL of a 0.5 M ferric nitrate solution (Fe(NO_3_)_3_·9H_2_O) at a fixed rate of 1 mL min^–1^ under constant stirring with a propeller, and under a steady stream of N_2_(g) to minimize contamination with dissolved (bi)carbonate species. The resulting slurry was aged at 60 °C for 72 h in an oven. Next, the precipitate was dialyzed (Spectra/Pore membrane 2) with Milli-Q water. The water was changed every day until its conductivity was close to that of Milli-Q water. The dialyzed suspensions were then stored in polypropylene containers at 4 °C until further use. X-ray diffraction (XRD) confirmed goethite as the sole crystallographic solid phase (Figure S1), and FTIR spectroscopy revealed only the main vibrational bands of goethite (Figure S2). The N_2_(g)-B.E.T. specific surface area of goethite was 89 ± 1 m^2^ g^–1^. The point of zero charge (PZC), previously determined by potentiometric titrations in 0.01, 0.1, and 1 M NaCl solutions at 298 K,^29^ was 9.1.

### 2.3 Batch Adsorption Experiments

Aqueous suspensions of 50 m^2^/L goethite in 10 mM NaCl were equilibrated at 25 °C with PA (0–200 μM) with a range of silicate concentrations (0–1 mM). The pH was maintained to the desired value (4.0 ≤ pH ≤ 10.0) by adding small volumes of 0.1 M HCl or NaOH. The samples were then equilibrated on an end-to-end rotator at 25 ± 1 °C for 1 or 30 days. These two reaction times were chosen based on previous time-resolved adsorption experiments,^13^ which revealed an initial rapid uptake of silicate and then substantially slower sorption rates. All suspension pH values were measured again before filtration (0.2 μm filter paper) with a benchtop pH/mV meter (HI2211, HANNA Instrument) calibrated on a daily basis and with a resolution of 0.01 pH. Final PA concentrations were analyzed by UV–vis spectrophotometry (Cary 5G UV–vis–NIR), and soluble silicate was determined using the molybdenum blue spectrophotometric method (detection limit of 1 μM).^52^ All experiments were repeated at least twice, with uncertainties in surface loadings of ±5% for PA and ±3% for silicate.

### 2.4 FTIR Spectroscopy

FTIR spectra of bound silicate and PA species were collected on N_2_(g)-dried goethite powder after reaction in aqueous suspensions of 50 m^2^/L goethite with PA and silicate at pH 4.0 or 6.0 for 1 day. The powder was produced by drying the centrifuged wet pastes of goethite under a stream of N_2_(g) (200 sccm, square cubic centimeter per minute) directly on an Attenuated Total Reflectance (ATR; diamond, single bounce; Golden Gate by Specac) cell for FTIR measurements. FTIR spectra were collected during the drying period until all O–H stretching (∼3400 cm^–1^) and bending (∼1630 cm^–1^) modes of free water disappeared. FTIR spectra were continuously collected in situ with a Bruker Vertex 70/V FTIR spectrometer equipped with a DLaTGS detector. All spectra were collected in the 600–4000 cm^–1^ range at a resolution of 4.0 cm^–1^, and at a forward/reverse scanning rate of 10 Hz. Each spectrum was an average of 250 scans, all taken over the course of 218 s.

### 2.5 Multivariate Curve Resolution Analysis of FTIR Spectra

Spectral components representative of distinct molecular species of sorbed PA were extracted from the 1300–1900 cm^–1^ region of spectra of goethite samples equilibrated over a range of PA surface loadings at pH 4 and 6. This chemometric analysis, built on Multivariate Curve Resolution Alternating Least Square (MCR-ALS),^53^ was implemented with a new code written for this study using MATLAB 2016b (MathWorks, Inc.). As in MCR-ALS,^53^ we applied the Beer–Lambert law (**A**_*m × n*_ = **ε**_*m × k*_**C**_*k × n*_) to extract *k* spectral components (**ε** ≥ 0) and their correlated concentrations (**C**_*k × n*_ ≥ 0) from a matrix of experimental absorbance (**A**_*m × n*_) data collected over *m* wavenumbers and *n* samples. In essence, the program iteratively rotates the first *k* orthogonal singular vectors (cf. eigenvectors) into a real chemical space conforming to these constraints (**ε**_*m × k*_ ≥ 0, **C**_*k × n*_ ≥ 0) to minimize the sum of squares of the deviation of the model from the experimental **A** data. In our new implementation of this approach, however, we selectively fixed **ε** values of PA species in noncompetitive systems to search for new species in the competitive Si-bearing system. This approach ensured that the inherent spectral profiles of PA species were not conflated with those of new, or poorly resolved, species.

### 2.6 Surface Complexation Modeling

The multisite complexation (MUSIC) model^54^ and the geochemical speciation code PHREEQC (version 2)^55^ were used for surface complexation calculations. The charge of the goethite/water interface was treated using the three-plane model (TPM) for the electric double layer (EDL). Charges of the adsorbates were distributed among the 0- (H^+^, metal-bonded PA and silicate), 1- (hydrogen-bonded PA and non-bonded portions of silicates), and 2- (Na^+^, Cl^–^) planes. Singly (≡FeOH^–0.5^; −OH), doubly (≡Fe_2_OH; μ-OH), and triply (≡Fe_3_OH^+0.5^; μ_3_-O(H)) coordinated oxygens outcrop the goethite surface, depending on the crystal face (Figure 1) and pH. The protonation of these species was predicted using a simplified 1-pK model, here neglecting the contributions of doubly and part of the triply coordinated oxygens. The reactive site density in this model was detailed in our previous work^28,29^ and includes 3.12 sites nm^–2^ of ≡FeOH^–0.5^ and 3.12 sites nm^–2^ of ≡Fe_3_O^–0.5^ on the (101) plane (90% of the surface area). Additionally, it includes 7.4 sites nm^–2^ of ≡FeOH^–0.5^ on the (210) plane (10% of the surface area). These goethite faces pertain to the *Pmab* space group. Equilibrium constants of all surface species are reported in Table 1.

**Table 1:** Surface Complexation Modeling Parameters

aqueous solutions^a^	log *K*				
PA^–2^ + H^+^ ⇌ PAH^–^	5.408				
PA^–2^ + 2H^+^ ⇌ PAH_2_	8.358				
H_4_SiO_4_ ⇌ H_2_SiO_4_^–^ + H^+^	–9.82				
H_4_SiO_4_ ⇌ H_3_SiO_4_^–2^ + 2H^+^	–23.27				
2H_4_SiO_4_ ⇌ Si_2_O(OH)_6_ + H_2_O	–1.5				
2H_4_SiO_4_ ⇌ Si_2_O_2_(OH)_5_^–^ + H^+^ + H_2_O	–8.5				

surface reactions	log *K*	Δ*z*_0_	Δ*z*_1_	Δ*z*_2_	ref
≡Fe_3_O^–0.5^ + H^+^ ⇌ ≡Fe_3_OH^+0.5^	9.1	+1	0	0	(28)
≡Fe_3_O^–0.5^ + H^+^ + Cl^–^ ⇌ ≡Fe_3_OH^+0.5...^Cl^–^	8.1	+1	0	–1	(28)
≡Fe_3_O^–0.5^ + Na^+^ ⇌ ≡Fe_3_O^–0.5...^Na^+^	–1	0	0	+1	(28)
≡FeOH^–0.5^ + H^+^ ⇌ ≡FeOH_2_^+0.5^	9.1	+1	0	0	(28)
≡FeOH^–0.5^ + H^+^ + Cl^–^ ⇌ ≡FeOH_2_^+0.5...^Cl^–^	8.1	+1	0	–1	(28)
≡FeOH^–0.5^ + Na^+^ ⇌ ≡FeOH^–0.5...^Na^+^	–1	0	0	+1	(28)
2H^+^ + 2≡FeOH^–0.5^ + PA^–2^ ⇌ (≡Fe)_2_(PA)^−^ + 2H_2_O	14.8	0	0	0	this study
2H^+^ + 2≡FeOH^–0.5^ + PA^–2^ ⇌ (≡FeOH_2_)_2_PA ^–^	19.5	+2	–2	0	this study
2≡FeOH^–0.5^ + H_4_SiO_4_ ⇌ (≡FeO)_2_HSiO_2_^–2^ + H^+^ + 2H_2_O	5.85^b^ (6.65)	0.48	–0.48	0	(8)
2≡FeOH^–0.5^ + 4H_4_SiO_4_ ⇌ (≡FeO)_2_SiHSi_3_O_3_(OH)_9_^–^ + 4H_2_O	13.89^b^ (14.69)	0.29	–0.29	0	(8)
2≡FeOH^–0.5^ + 4H_4_SiO_4_ ⇌ (≡FeO)_2_SiHSi_3_O_4_(OH)_8_^–2^+ H^+^ + 4H_2_O	6.6^b^ (8.27)	0.29	–1.29	0	this study

a Log *K* values for aqueous solutions are from the minteq.v4 database in PHREEQC (version 2).^55^

b Log *K* values for 1 day reaction time (values for 30 days are provided between parenthesis).

## 3 Results and Discussion

**Figure 2:**
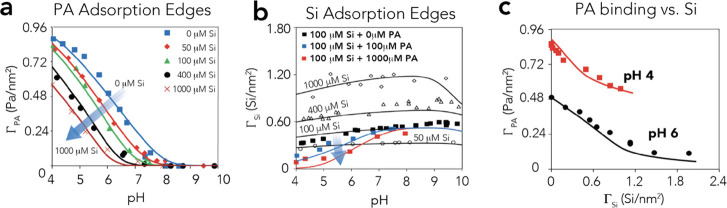
Phthalate (Γ_PA_; PA species per nm^2^) and silicate (Γ_Si_; Si^4+^ per nm^2^) loadings achieved after 1 day of reaction in 50 m^2^/L goethite suspensions in 10 mM NaCl at 298 K. (a) PA adsorption edges in goethite suspensions of 100 μM PA with 0–1000 μM silicate. (b) Silicate adsorption edges in separate (0–1000 μM silicate) and competitive (0–1000 μM PA) systems. (c) PA loadings at pH 4 and 6 in relation to bound silicate obtained from suspensions with 100 μM PA and 0–1000 μM silicate. All lines are predictions are from the SCM of this study. Γ denotes surface loadings in terms of PA species per nm^2^ (Γ_PA_) and Si^4+^ per nm^2^ (Γ_Si_).

Silicate competitively binds with PA below pH ∼8, where PA is adsorbed (Figure 2a,b). This occured at silicate concentrations representative of natural waters (50–1000 μM). PA adsorption edges were, as a result, effectively shifted to lower pH values (Figure 2a), and were associated to the loss of ∼0.3 PA per sorbed silicate species at both pH 4 and 6 (Figure 2c).Silicate binding was, in turn, lowered below pH ∼8 from the competition of PA binding for reactive −OH groups (Figure 2b). We also note that the sum of PA (Γ_PA_, PA/nm^2^) and (mono- or polymeric) silicate loadings (Γ_Si_, Si^4+^/nm^2^) never exceeded the density (3.5 sites/nm^2^) of reactive −OH groups involved in PA and silicate binding (Figure 1).

### 3.1 Modeling

To develop a predictive model for this competitive system, we recalibrated literature^8,17,30,31^ SCM models for silicate and PA binding on goethite using the precise solution conditions, and surface complexation modeling framework chosen for this study. This approach was necessary to ensure that any thermodynamic predictions and molecular-based interpretations from subsystems were accurate for the multicomponent, competitive system, under study. Therefore, we begin by briefly describing these models, as they are needed to aid our discussion of competitive silicate and PA binding.

**Figure 3:**
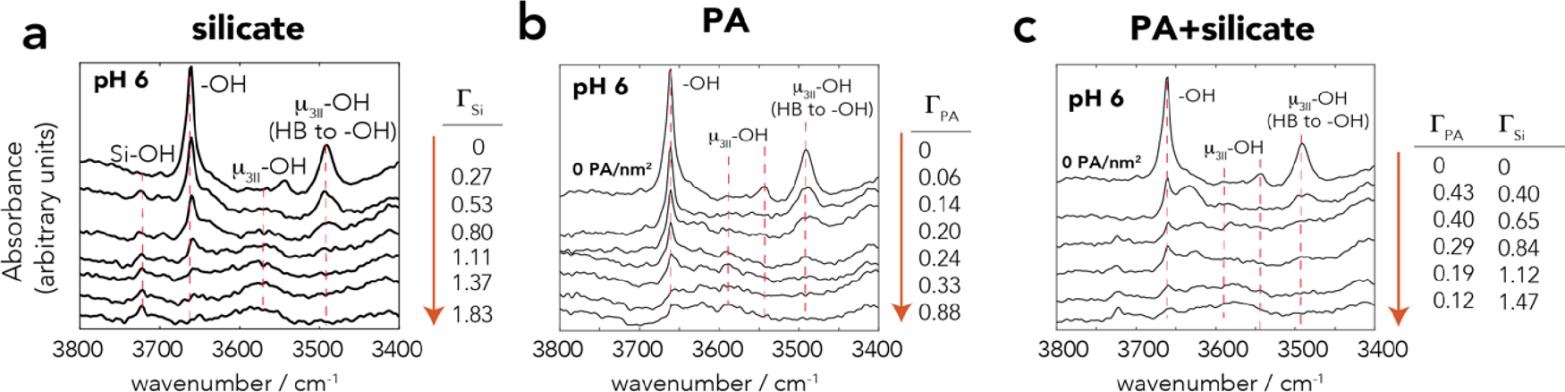
FTIR spectra of the O–H stretching region of the surface OH group and of Si–OH groups at the goethite surface. The spectra were obtained after 1 day of reaction with (a) silicate, (b) PA, and (c) silicate and PA in aqueous suspensions of 50 m^2^/L goethite in 10 mM NaCl at 298 K by drying centrifuged wet goethite pastes with a flow of N_2_(g) on the ATR cell at 298 K. Here, the dominant 3661 cm^–1^ band of the exchangeable −OH group was removed by PA and silicate binding. The loss of −OH altered the hydrogen bonding environment of unreacted −OH groups, and removed pre-existing accepting hydrogen bonds from neighboring μ_3_-OH sites (Figure 1). Si–OH groups of bound silicate species were detected at ∼3710 cm^–1^. All band intensities were normalized from the bulk O–H stretching band of goethite at 3120 cm^–1^ (Figure S2). Γ denotes surface loadings in terms of PA species per nm^2^ (Γ_PA_) and Si^4+^ per nm^2^ (Γ_Si_).

To predict silicate binding, we used our recent silicate-binding model (Table 1)*.*^21^ The model captures the broad pH and strong binding affinity of silicate on goethite (Figure 2b and Figure S3).^8,12,14^ Building on Kanematsu et al*.*,^13^ we were able to use the model to predict silicate binding by a ligand exchange reaction with −OH sites (Figure 1). These binding modes align with new sets of FTIR spectra (Figure 3a for pH 6 and Figure S4 for pH 4) of dried samples, revealing the preferential consumption of their signature 3661 cm^–1^ band alongside the disruption of hydrogen bonds with the neighboring μ_3II_-OH group (3490 cm^–1^). These spectra also showed that the resulting silicate surface complex exposed Si–OH groups through another signature band at ∼3720 cm^–1^. As Kanematsu et al.^13^ also suggested that monomeric silicate complexes form a hydrogen bond with the neighboring −OH site (Figure 1), we modeled silicate binding using the reaction (log *K*(≡FeO)_2_Si(OH)_2_^–^).
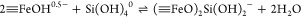
1

**Figure 4:**
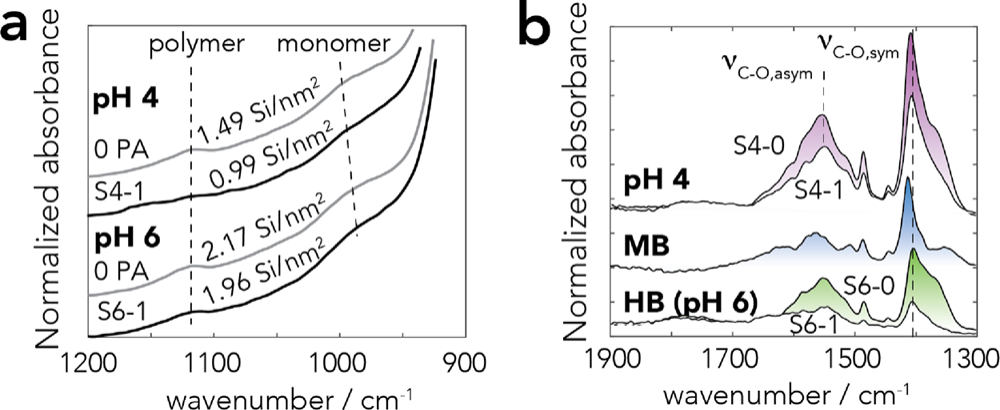
FTIR spectra of the (a) Si–O stretching region and (b) C–O stretching region of centrifuged wet goethite pastes at pH 4 and 6 in noncompetitive (0 PA = 0 PA/nm^2^; S4-0 = 0.86 PA/nm^2^; S6-0 = 0.50 PA/nm^2^) and competitive (S4-1 = 0.55 PA/nm^2^ and 0.99 Si^4+^/nm^2^; S6-1 = 0.11 PA/nm^2^ and 1.96 Si^4+^/nm^2^) systems. All spectra were obtained after 1 day of reaction in aqueous suspensions of 50 m^2^/L goethite in 10 mM NaCl at 298 K. All intensities were normalized for the bulk Fe–O–H bending region (Figure S2). To obtain spectral profiles of PA only in (b), (i) the bending band of liquid water (∼1630 cm^–1^) was removed from the spectrum of wet unreacted goethite and (ii) the combination (1662 cm^–1^) and overtones (1789 cm^–1^) of Fe–O–H bending modes were removed from the spectra of dry goethite (cf. Figure S8 for uncorrected spectra). The spectral profile of HB species was obtained from the spectrum at pH 6, and the spectral profile of MB species was obtained by subtracting the spectrum at pH 4 from the spectrum at pH 6.

Therefore, this reaction aligns with previous models for a bidentate surface complex,^8,17^ although it uses a charge distribution scheme (Table 1) for a hydrogen-bonded monodentate inner-sphere complex. The model also accounts for polymeric species to predict binding at greater silicate loadings, for example, those exceeding ∼1.5 Si^4+^ per nm^2^ where additional silicate loadings did not decrease PA loadings (Figure 2c).^32^ These polymeric species were detected by FTIR spectroscopy (Figure 4a). For practical modeling reasons,^8^ these species were expressed solely through tetrameric species with (log *K*(≡FeO)_2_SiHSi_3_O_3_(OH)_9_^–^) and (log *K*(≡FeO)_2_SiHSi_3_O_4_(OH)_8_^–2^):
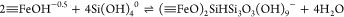
2
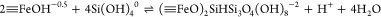
3

The model predicts polymeric species at silicate loadings exceeding ∼0.9 Si^4+^/nm^2^ at pH 4 and ∼1.1 Si^4+^/nm^2^ at pH 6 (Figure S5). This is supported further by FTIR spectroscopy^13^ where the Si–O band characteristic of Si–O–Si linkages appears in samples where bands of −OH groups were already consumed (Figure S3). We also note that, from an electrostatic standpoint (Figure S6), silicate binding lowered the outer-Helmholtz potentials by ∼0.03–0.04 V throughout the entire pH range and lowered the point of zero charge from 9.1 to 8.3. Silicate binding therefore intrinsically weakened the electrostatically driven binding of organic species on goethite.

PA binding was predicted using a recalibration of our previous SCM model (Table 1) involving hydrogen-bonded (HB) and metal-bonded (MB) species (Figure 1).^30,31^ FTIR spectra confirmed our previous findings^30,31^ supporting the predominance of HB complexes at pH 6 and the coexistence of both complexes at pH 4 (Figure 4b and Figures S7 and S8). HB species formed direct hydrogen bonds between carboxyl groups and surface OH groups, and MB complexes formed direct Fe–carboxylate bonds after ligand exchange with OH groups (Figure 1). The primary involvement of (singly coordinated) −OH groups in the formation of these species was confirmed by FTIR of dried goethite through the preferential loss of the signature 3661 cm^–1^ band of −OH (Figure 3b for pH 6 and Figure S4 for pH 4) with PA loadings. These spectra also revealed that the remaining unreacted −OH groups were more strongly hydrogen-bonded. This can be appreciated by a broad and low-lying band at lower O–H stretching frequencies (∼3575–3650 cm^–1^) appearing at high PA loadings (Figure 3b). Ideally, the steric constraints at the dominant (101) plane should promote bridging between two Fe atoms separated by ∼3 Å from one another (Figure 1),^33^ while at the (210) plane two ≡FeOH^–0.5^ should be located on the same Fe(III) octahedron. As such, we modeled PA binding as a 1:2 PA/≡FeOH^–0.5^ species as follows:
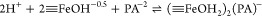
4
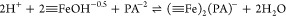
5Of note, the charge-distribution and capacitance values of the compact layers (Table 1) generated the pH-dependent loadings of these species^34^ (Figure S7c) and lowered outer-Helmholtz (1-plane) potentials by up to ∼0.1 V below pH ∼8 (Figure S6). Our recalibrated model (Figure 2 and Figure S6) predicts the pH- and concentration-dependent PA loadings (Table 1).^30^

**Figure 5:**
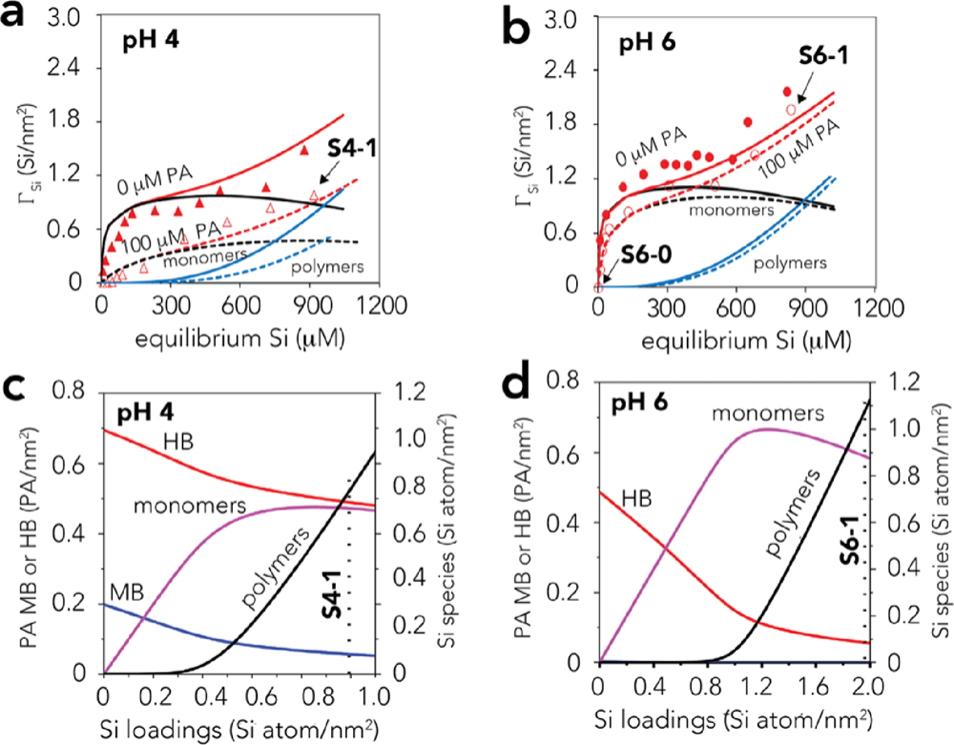
(a, b) Silicate adsorption isotherms and (c, d) speciation from competitive model predictions at pH 4 and 6. Conditions for samples S4-1 and S6-1, for which the FTIR spectra are shown in Figure 4b, are indicated by (a, b) the arrow pointing to data points and (c, d) the vertical dotted lines.

**Figure 6:**
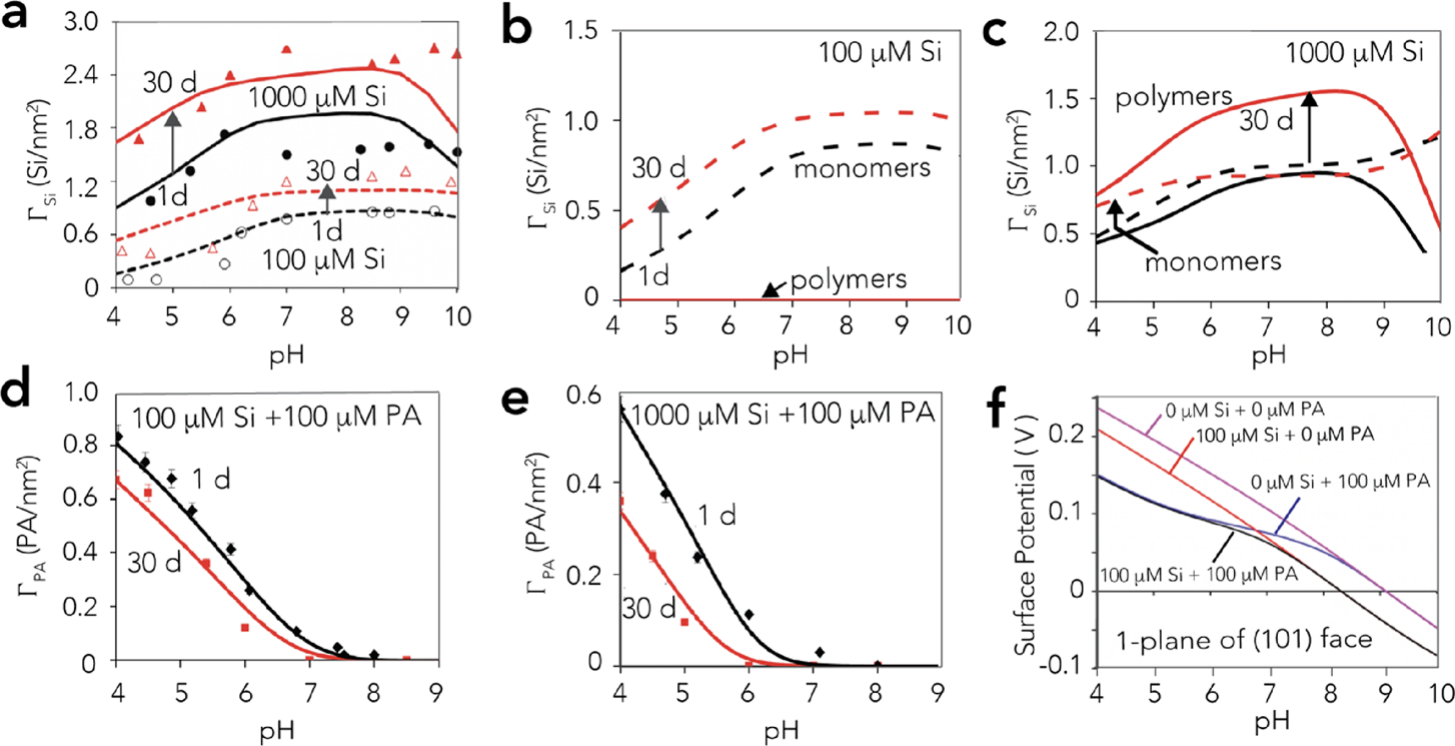
Batch adsorption data and SCM modeling. (a–c) Silicate loadings, (d, e) PA loadings, and (f) surface electrostatic potentials achieved in suspensions of 50 m^2^/L goethite in 10 mM NaCl at 298 K. (a–e) Comparison of experimental and predicted SCM loadings achieved at 1 day (black) and 30 days (red) of reaction time in competitive systems with total concentrations of 100 μM PA with (a, b, d) 100 or (a, c, e) 1000 μM silicate. (f) Predicted surface potentials of the 1-plane of the (101) face of goethite containing unbound silicate oxo groups and carboxyl groups of HB PA species (Table 1; cf. Figure S6 for full modeling results).

Using these subsystem models, we explored a new model for the competitive binding of PA and silicate on goethite. This search involved various hydrogen-bonded interactions between coexisting PA and silicate complexes bound to vicinal (exchanged) −OH groups. However, the simplest and most accurate model predicting the adsorption data (Figures 2, 5, and 6) was generated from the subsystem model predictions alone. As such, the model predicts PA and silicate binding in terms of (i) the competition for −OH groups without involving PA/silicate interactions and (ii) a concomitant lowering of positive 1- and 2-plane electrostatic potentials by these anions (Figure 6f and Figure S6). The competition for −OH groups aligns with the O–H stretching region of goethite (Figure 3c), chiefly revealing the same spectral features as in the subsystems (Figure 3a,b). However, we note that nearly equimolar loadings of PA and silicate (Γ_PA_ ≈ Γ_Si_ ≈ 0.4 species/nm^2^) red-shifted the 3661 cm^–1^ band to ∼3640 cm^–1^. Although this idea needs further support (*e.g.*, by molecular modeling), this newly resolved band could indicate a regular spatial distribution of PA and silicate species along rows of exchanged and unreacted −OH groups (Figure 1).

The model thus predicted PA removal by the concomitant loss of both HB and MB species under acidic conditions and by the loss of HB species at circumneutral pH (Figure 5c,d). At pH 4, the model predicted no clear preferential removal of HB species over MB species (Figure 5c) as silicate binding also targeted the same −OH groups needed for PA species formation. Speciation changes predicted by the model also align with FTIR spectra of wet goethite pastes at pH 4 and 6 (Figure 4b). There, we found that decreased band intensities of PA species associated with silicate uptake did not alter the spectral profiles. Most notably, the symmetric C–O stretching band of PA (ν_CO,sym_) remained centered at 1408 cm^–1^, while a preferential removal of HB over MB species would have shifted this band to 1412 cm^–1^ (Figure S8).^31^

Model predictions also involved polymeric silicate species to explain additional loadings achieved at the greatest concentrations under study and where PA loadings remained unchanged (*e.g*., Figure 2c). Polymeric species appeared at silicate loadings exceeding ∼0.4 Si^4+^/nm^2^ at pH 4 and ∼0.8 Si^4+^/nm^2^ at pH 6 (Figure 5c,d). The conditions correspond to dissolved silicate concentrations of ∼300 μM (Figure 5a,b). Accordingly, the Si–O stretching region (Figure 4a) revealed coexisting monomeric and polymeric species. The model even aligns with the apparent decrease in polymeric species at pH 4 (S4-1; Figure 4a) resulting from the competitive binding of MB PA species, and with relatively unchanged polymer loadings at pH 6 (cf. Figure 2c).

### 3.2 Aging

To investigate further the potential impact that silicate polymers could have on PA loadings, we used our model to predict loadings achieved after 30 days of reaction time (Figure 6). This reaction time was chosen based on our previous work^13^ showing the development of silicate polymeric species on goethite. These efforts at two environmentally relevant silicate concentrations (100 and 1000 μM) revealed larger silicate loadings over the entire pH range (pH 4–10) without, however, any substantial changes in the shape of the adsorption envelope (Figure 6). For example, the maximum silicate loadings increased from ∼0.9 to ∼1.3 Si^4+^/nm^2^ for 100 μM silicate and from ∼1.6 to ∼2.7 Si^4+^/nm^2^ for 1000 μM silicate solutions reacted with 50 m^2^/L goethite (Figure 6). The most effective and simple approach to account for these loadings was to raise all silicate binding constants by 0.8–1.7 log *K* unit from the values that we obtained at 1 day reaction time (Figure 2 and Table 1). The resulting model predicted a ∼2.3-fold increase in monomeric and ∼1.8-fold increase in polymeric species after 30 days of reaction time and therefore reflects the longer-term reaction needed for silicate binding on goethite. In this sense, we treat binding of all other species to be in dynamic equilibrium in relation to silicate binding. Accordingly, the model predicts that solutions of 100 μM silicate only decreased PA loadings when monomeric species increased. However, the formation of polymeric species was more significant in solutions of 1000 μM silicate. Because greater concentrations of these species consumed an even greater number of reactive sites (cf. chemical speciation in Figure 6c and Figure S5) and because they lowered electrostatic potentials (Figure 6f), PA loadings were also lowered.

### 3.3 Drying

**Figure 7:**
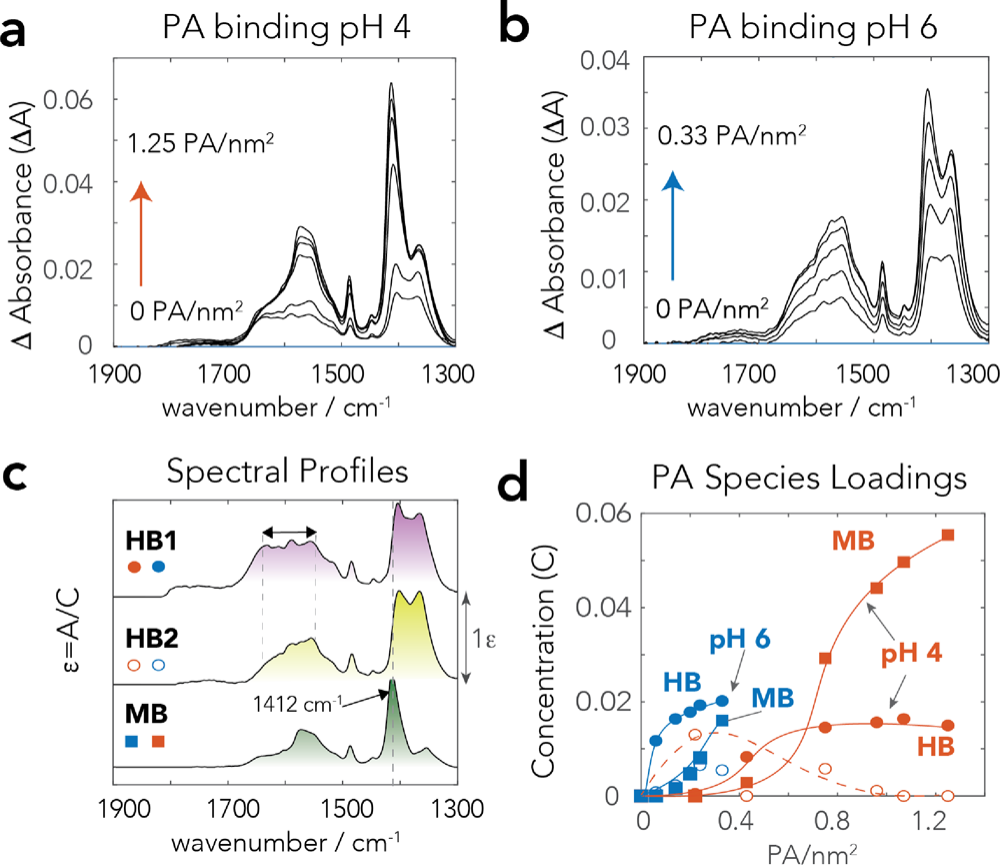
Net ATR-FTIR spectra of PA bound to N_2_-dry (298 K) goethite with removed contributions from overtones and combinations of goethite bulk bending modes (cf. Figure S8 for uncorrected spectra). Samples were initially reacted in 50 m^2^/L suspensions of goethite in 10 mM NaCl at 298 K with 0–800 μM PA at pH (a) 4 and (b) 6 for 1 day and then dried under a stream of N_2_(g). Increased loadings are isotherms at these fixed pH values. Chemometric (MCR) analyses decomposed these spectra (*A*) into (c) ε (n.b. normalized arbitrary scale) and (d) concentration (*C*) profiles shown in (b) such that *A* = ε × *C*. Lines in (d) are only visual guides to the data for which the legends are shown in (c).

Finally, to explore the fate of these coexisting PA and silicate species exposed to drying, we studied the FTIR spectral profiles of dehydrated goethite particles previously reacted for 1 day in mixed aqueous solutions of PA and silicate (Figure 7). To assist this task, we first identified the predominant molecular species of PA produced by drying. A chemometric analysis with these new FTIR spectra (Section 2.5) showed that HB and MB species persisted to the dry state. However, the removal of water facilitated the formation of MB over HB species, as can be appreciated by the PA species loadings shown in Figure 7d (See Figure S7 for comparison with speciation in contact with liquid water). This analysis extracted two forms of HB (HB1 and HB2) species, mostly with different breadths in the asymmetric C–O stretching (ν_CO,asym_) region. These differences relate to differences in inhomogeneous band broadening caused by hydrogen-bonded interactions with surface OH groups of contrasting O–H bond strength. The species with the narrower ν_CO,asym_ region (HB1) predominates only at low PA loadings, as PA likely targets a collection of surface OH groups with a narrower distribution of O–H bond strength (Figure 1). The consumption of these groups at larger PA loadings, however, favors interactions with other OH groups with a broader range of O–H bond strengths (HB2). These OH groups likely include μ-OH and various μ_3_-OH groups of the goethite surface (Figure 1).

**Figure 8:**
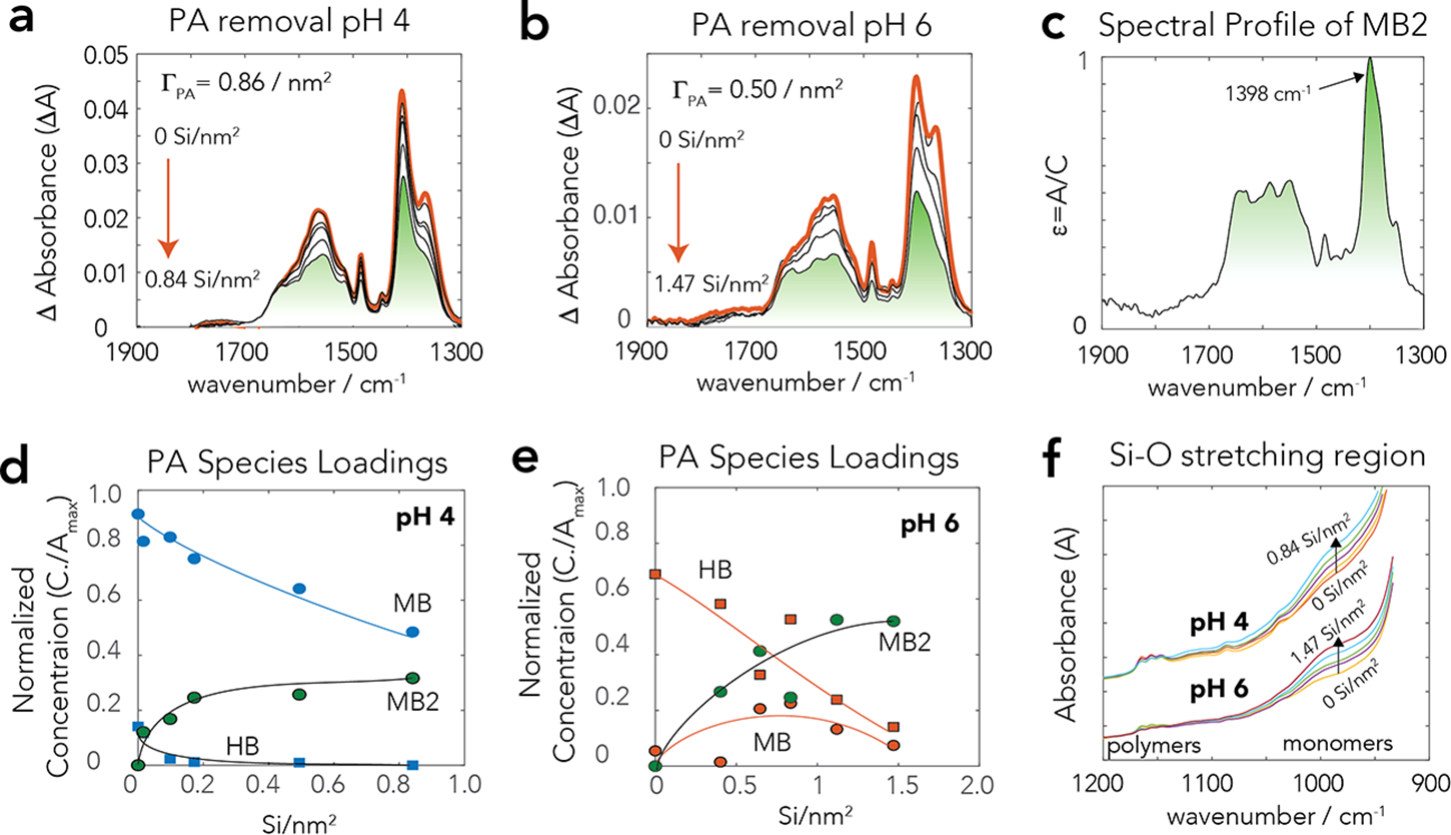
ATR-FTIR spectra and chemometric (MCR) analyses of N_2_-dry (298 K) goethite initially reacted in 50 m^2^/L suspensions in 10 mM NaCl at 298 K (cf. Figure S8 for uncorrected spectra). (a, b) Spectra at pH (a) 4 and (b) 6 at various PA loadings with 100 μM PA and 0–1000 μM Si. Note that combination (1662 cm^–1^) and overtones (1789 cm^–1^) of Fe–O–H bending modes (Figure S8) were removed. Decreased PA loadings result from the competitive binding of silicate at these fixed pH values. (c–e) Chemometric analyses used concentration profiles of HB and MB of Figure 7 to also extract (c) the spectral profile of a new component, PA–Si. The associated concentration profiles of these three species reveal the predominant removal of (d) MB species at pH 4 but of (e) HB species at pH 6. Lines in (d) and (e) are only visual guides. (f) The Si–O stretching region in corresponding spectra of (a) and (b) showing the preferential appearance of monomeric silicate species.

As in our batch adsorption data (Figure 2), FTIR spectra of dry goethite at pH 4 and 6 (Figure 8a,b) after reaction in the competitive system reflected the systematic loss of PA loadings resulting from silicate binding. A chemometric analysis of the C–O stretching region extracted only one dominant HB PA species comparable to HB2 (Figure 7) but now with a new MB-like species (MB2), with the spectral profile shown in Figure 8c. The new MB2 species replaced up to ∼1/3 of the original MB species at pH 4. At pH 6, however, the absence of water facilitated the replacement of HB by MB2, and by a more minor fraction of MB species the previously coexisting MB and HB1/HB2 species at pH 6 (Figure 8d,e). We contend that the loss of water broadened the ν_CO,asym_ region of MB2 over a considerably larger range of frequencies than in MB (Figure 7c). This silicate-induced inhomogeneous band broadening could thereby suggest that MB2 species interacted with OH groups with a broader range of O–H bond strengths/acidities than on dry goethite in the absence of silicates (Figure 7). This spectral profile may even possibly arise from interactions with neighboring unreacted goethite hydroxo, and with Si–OH groups of co-sorbed silicate species.

These results consequently showed that drying facilitated the formation of MB species of PA in the presence of bound silicate. These bound silicates remained predominantly in the form of monomeric species when loadings were at least up to 1.12 Si^4+^/nm^2^ (Figure 8f). Additional polymeric species did not appear because of the low concentrations of unbound silicate species in the wet goethite pastes prior drying. As such, our finding that dry goethite predominantly exposed coexisting MB PA species with monomeric silicate species even aligns with our SCM model of wet goethite pastes from which these products were made. We find these results encouraging for pursuing future studies dedicated to bridging the speciation of wet and dry interfacial systems, which are becoming crucial for understanding how wet/dry cycling impacts competitive binding at mineral surfaces.

## 4 Conclusions

This batch adsorption, ATR-FTIR spectroscopy, and surface complexation modeling study revealed the pH-, concentration-, and time-dependent competitive binding of silicate ions and PA on goethite. Our surface complexation model can now adequately predict competitive binding for reactive −OH functional groups of the goethite surface only using formation constants of HB/MB PA species and monomeric/polymeric silicate species obtained from subsystems and whose presence was assessed by ATR-FTIR spectroscopy. The model can also account for the more effective competition of silicate species after 30 days of reaction time by a greater concentration of monomeric silicate species at low (100 μM) and of polymeric species at high (1000 μM) silicate concentrations. It also provides an adequate description of silicate and PA loadings over a wide range of concentrations, and pH values.

Dehydration altered the molecular scale speciation of coexisting PA and silicate species. While both HB and MB species of PA persisted under the dry state, the removal of water favored MB over HB species. These findings are important for understanding the (geo)chemistry of unsaturated environments subject to water fluctuations, such as vadose zones of soils. As MB species bind more strongly to minerals than HB species, drying soils could facilitate the attachment of organics at mineral surfaces and therefore decrease the transport of organic contaminants in aquatic environments. In addition, drying could facilitate silicate polymerization and therefore the reactivity of minerals,^13^ decreasing the quantity of contaminants adsorbed by minerals. These findings consequently call for new models over a broader range of carboxylate-bearing organic contaminants to understand their fate in soils, especially those undergoing wet–dry cycles. In particular, future studies on the wettability of carboxylate- and Si-coated minerals and rehydration reactions of, for example, MB organic species triggered by dehydration may be especially important for understanding the fate of organic contaminants in nature.

## Supporting Information Available

Goethite X-ray diffractogram; goethite FTIR spectra; silicate binding edges and Si–O and O–H stretching bands of corresponding species; thermodynamic modeling of monomeric and polymeric silicate species: PA adsorption edges and corresponding thermodynamic modeling; and FTIR spectra of bound PA (PDF)
