# Supplementary material for: Competitive Carboxylate–Silicate Binding at Iron Oxyhydroxide Surfaces
Source: Langmuir. 2021 Oct 29;37(44):13107–15. doi: 10.1021/acs.langmuir.1c02261 (PMC8582244; doi:10.1021/acs.langmuir.1c02261)
Supplement: Supplementary file 1 — la1c02261_si_001.pdf [file la1c02261_si_001.pdf]

SUPPORTING INFORMATION

Competitive Carboxylate-Silicate Binding at Iron Oxyhydroxide Surfaces

Wei Cheng,[a,b] Rémi Marsac,[c] Khalil Hanna,[a] Jean-François Boily[b]*

[a]Ecole Nationale Supérieure de Chimie de Rennes, CNRS, UMR 6226, 11 Allée de Beaulieu, 35708 Rennes Cedex 7, France.

[b]Department of Chemistry, Umeå University, Umeå, SE-901 87, Sweden.

[c]Géosciences Rennes UMR 6118, Université Rennes 1, CNRS, 35042 Rennes cedex, France.

*Corresponding author: jean-francois.boily@umu.se

Table of Contents

Figure S1..3
Figure S2..3
Figure S3..4
Figure S4..5
Figure S5..6
Figure S6..6
Figure S7..7
Figure S8..8
References..9

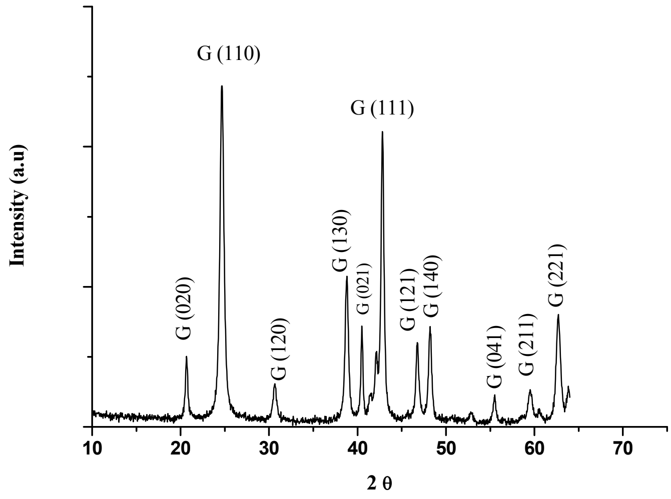

Figure S1. X-ray diffractogram of dry, unreacted, goethite used for this work. The diffractogram confirms that goethite was the sole crystallographic phase.

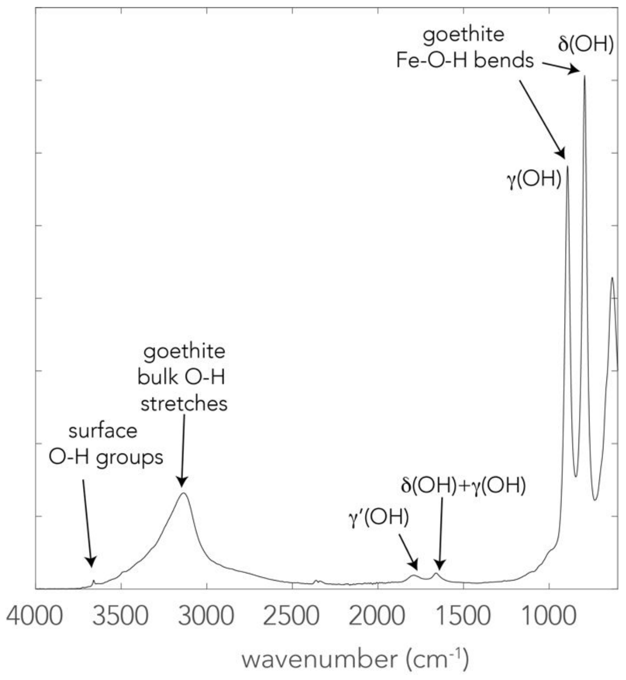

Figure S2. Fourier Transform Infrared (FTIR) spectrum of dry, unreacted, goethite used for this work. The spectrum confirms that no amorphous iron (oxy)(hydr)oxide co-exists with the sample. The bulk O-H stretching (ν(OH)) of goethite is at 3120 cm^{-1}, and is accompanied by a collection of surface OH groups at > 3500 cm^{-1}. The γ'(OH) band at 1789 cm^{-1} is the first overtone of the in-plane bending (deformation; γ(OH)) mode at 895 cm^{-1}, and the δ(OH)+ γ(OH) at 1662 cm^{-1} is the combination mode of γ(OH) and of the out-of-plane bending (deformation; δ(OH)) mode at 792 cm^{-1}).

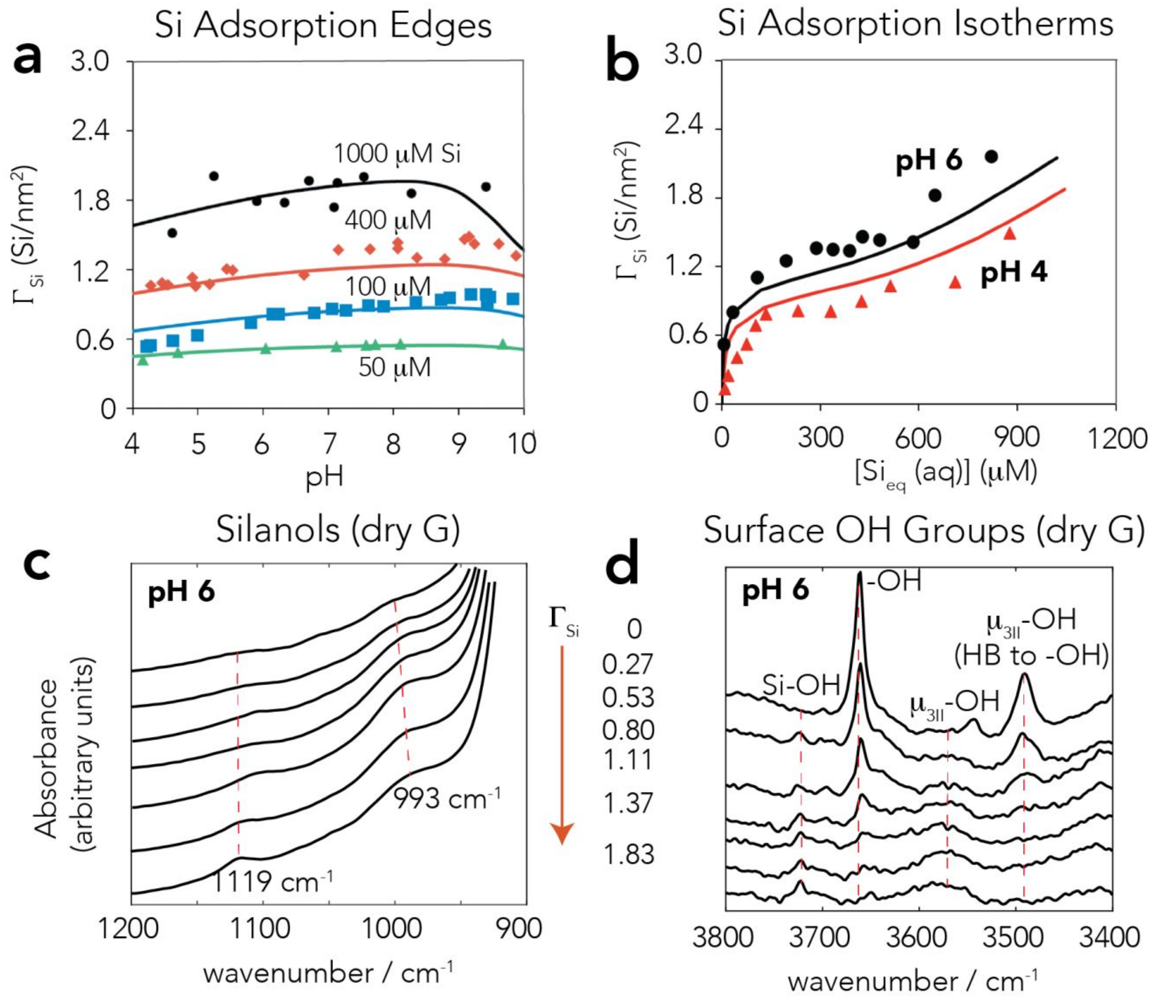

Figure S3. Silicate binding achieved after 1 d of equilibration in aqueous suspensions in 50 m^2/L goethite in 10 mM NaCl at 298 K. Adsorption (a) edges and (b) isotherms reporting Si loadings (Γ_{Si}) achieved from solutions of up to 1000 μM silicate, with lines resulting from our SCM. (c-d) Fourier Transform Infrared spectra of the (c) Si-O and (d) surface O-H stretching regions of goethite samples dried under N_2(g). The Si-O stretching region (c) reveals monomeric species at 993 cm^{-1} and Si-O-Si linkages at 1119 cm^{-1}. The surface O-H stretching region (d) reveals the preferential consumption of -OH groups by silicates, its impact on the hydrogen bond environment of the neighboring μ_{3II}-OH group, and the appearance of Si-OH groups from bound silicate species (Si-OH). More details on these findings, which we confirmed in this study, can be found in Kanematsu *et al.* [1] The symbol 'Γ' denotes surface loadings in terms of Si(IV) per nm^2 (Γ_{Si}).

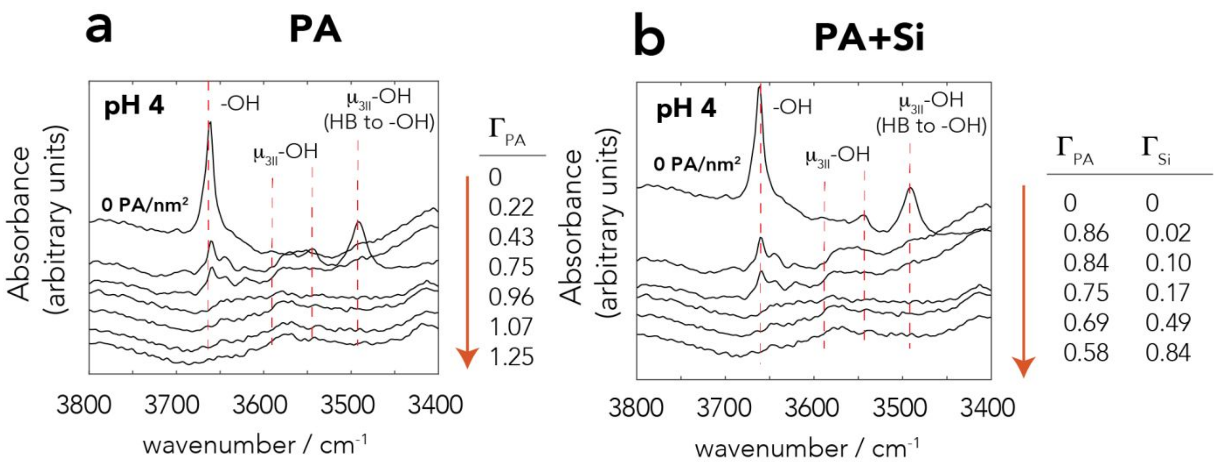

Figure S4. Fourier Transform Infrared spectra of the surface O-H stretching region of goethite samples dried under N_2(g). Samples were equilibrated for 1 d in aqueous suspensions in 50 m^2/L goethite in 10 mM NaCl at 298 K with (a) PA and (b) mixed PA and Si at pH 4. (a) PA consumes the same –OH sites as Si and also impacts of the hydrogen bond environment of the neighboring μ_{3II}-OH group. (b) In the mixed PA+Si system, both PA and Si compete for the same sites and split the –OH band in a distinctive way that in the subsystems. This splitting could possibly arise from altered hydrogen bonding environments.

n.b. From the study Kanematsu *et al.*[1] the O-H stretching region reveals that Si binding produced metal-bonded (MB) species with underlying Fe^{3+} sites form a ligand exchange reaction involving only singly-coordinated hydroxyl groups (–OH) of goethite (Figure 3). More specifically, these ligand exchange reactions can be appreciated by the loss of surface OH groups of goethite are with Si loading (Figure 3a),[1,2] a detailed account of these specific spectral signatures is given in a series of articles from our group (Figure 1).[1,3–7] Briefly, the important loss in intensity of the band generable by singly-coordinated −OH groups (3661 cm^{-1}) with Si loadings results from Si/OH exchange. In contrast, changes in the band positions of the resolvable μ_3–OH groups (*e.g.* shift from 3490 cm^{-1} band to ~3560 cm^{-1}) was rather the result of rupture or weakening of the original intersite $\mu_{3,II}$–OH···OH− hydrogen bond. [3,8] The losses of goethite surface functional groups correlate with the appearance of both Si-OH at 3720 cm^{-1} (Figure 3a) and Si-O bonds in the Si-O stretching region (Figure 3a). Combined with our batch adsorption data, these results thus point to a preferential loss of –OH groups for Si loadings of no more than Γ_{Si}=1.83 Si/nm^2. These loadings are well within the total –OH density of 3.5 sites/nm^2. From our conceptual model of the goethite surface (Figure 1), Si is likely bound as monodendate mononuclear silicate species along rows of these –OH sites on the dominant crystallographic faces of goethite (Figure 1).

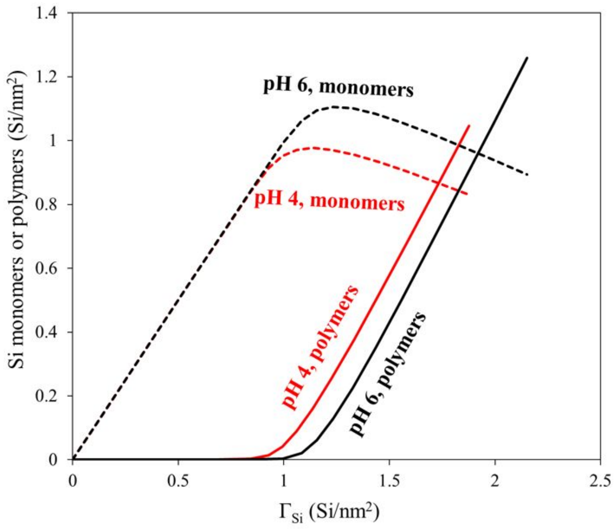

Figure S5. Speciation of Si monomers and polymers from SCM model predictions at pH 4 and 6.

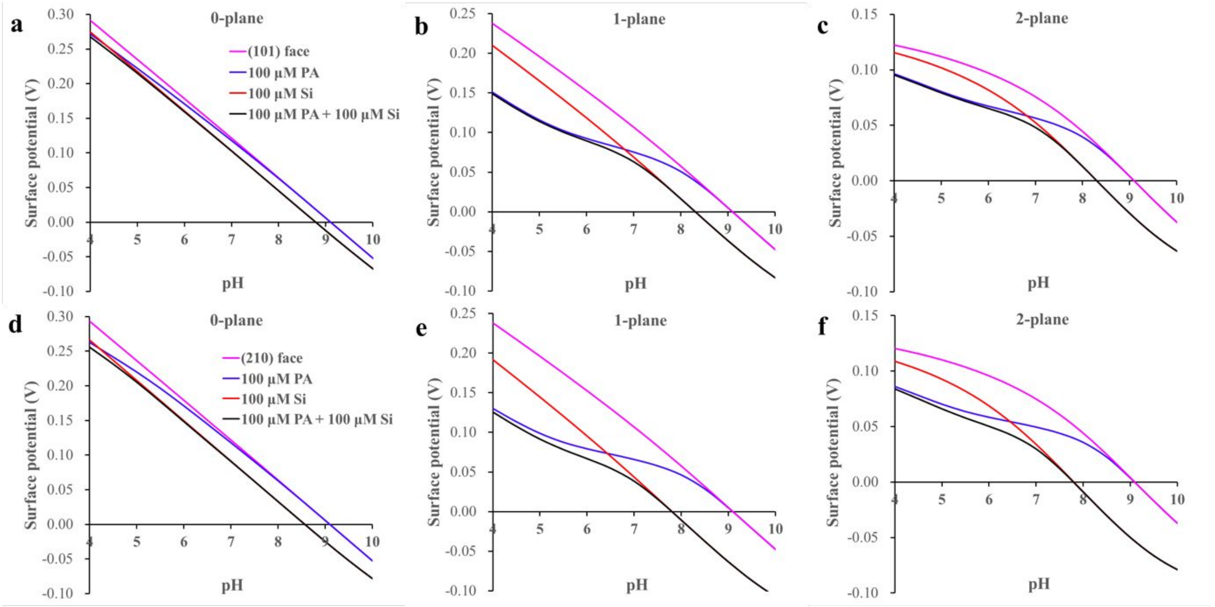

Figure S6. Predicted electrostatic potential of the goethite (a-c) (101) and (d-f) (210) faces for the (a,d) 0-, (b,d) 1- and (c,f) 2-planes of suspensions of 50 m^2/L goethite in 10 mM NaCl at 298 K. Pink lines are for the PA- and Si-free systems. Inner-Helmholtz potentials (0-plane) are sub-Nernstian with a slope of 52-54 mV/pH, which is characteristic for potential-determining H^+ and OH^- ions at insulating iron oxyhydroxide surfaces. Metal-bonding of PA lowers the slope at low pH where by displacing surface hydroxyl groups, while metal-bonding silicate binding lowers the apparent point of zero charge (9.1) as it binds over a wide range of pH values. Outer-Helmholtz potentials (1-plane) and diffuse layer (2-plane) potentials in the competitive systems are combinations of PA-dominated values at low pH and Si-dominated values at high pH. As such, the electrostatics contributions to the concomitant decrease in PA and Si loadings below pH 8 (*cf.* Fig. 2 of main text) relates to a lowering of the positive electrostatic potential of goethite by the co-existing anions.

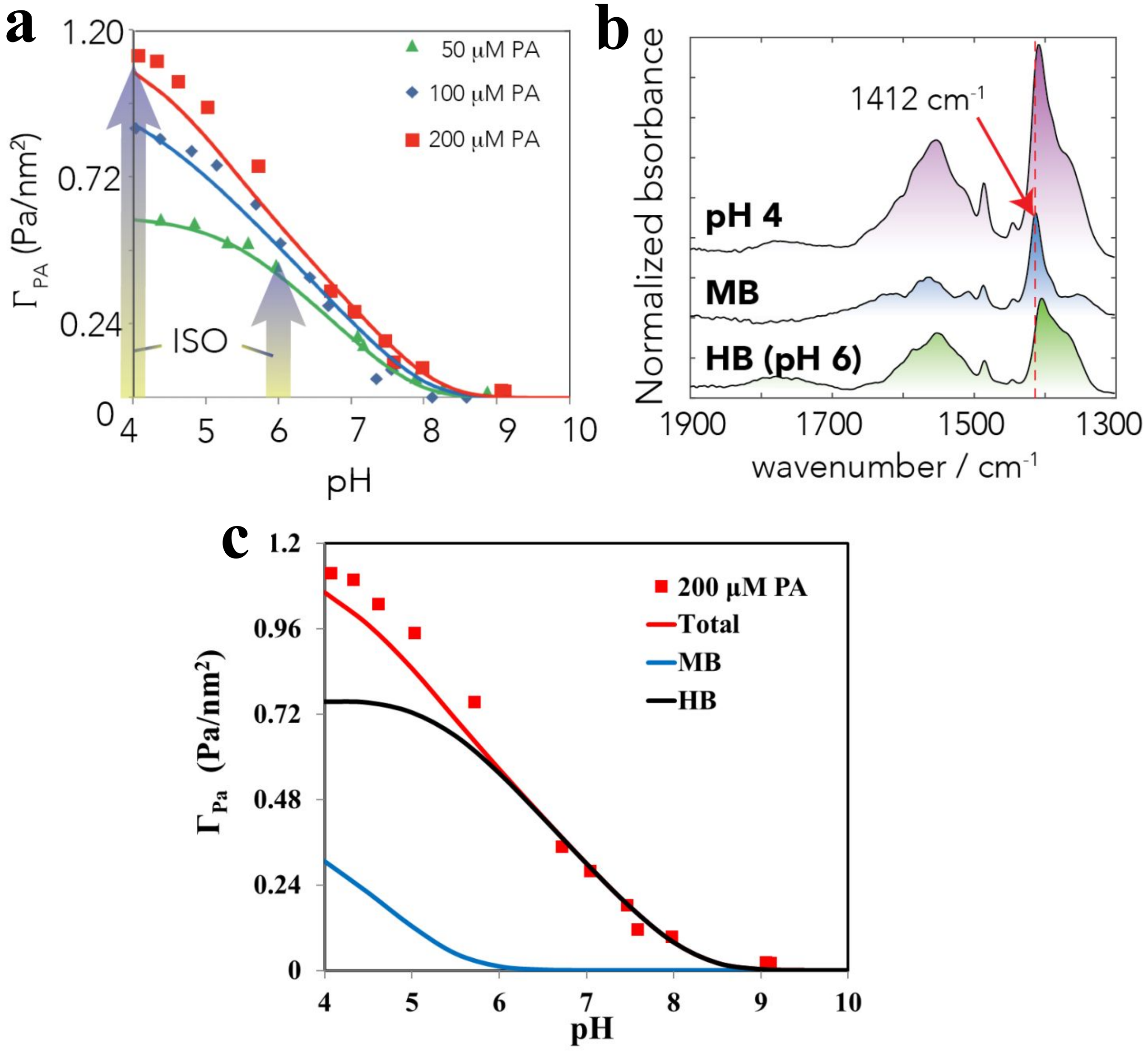

Figure S7. (a) Phthalate (Γ_{PA}) loadings achieved after 1 d of equilibration in aqueous suspensions in 50 m^2/L goethite in 10 mM NaCl at 298 K at 50, 100 and 200 μM PA. Lines are from the SCM adjusted for this work. Vertical arrows labeled 'ISO' show loading ranges explored in this system by FTIR (Figure 7 a-b). (b) ATR-FTIR spectra of sorbed PA at pH 4 and 6. MB and HB refer to metal- and hydrogen-bonded PA, respectively. Absorbance (A) values at pH 4 were subtracted from those at pH 6 (A pH 4 − A pH 6) to obtain an estimate of the spectral profile generated by MB complexes. (c) Predicted pH-depedence of hydrogen- (HB) and metal-bonded (MB) PA species at a total concentration of 200 μM PA.

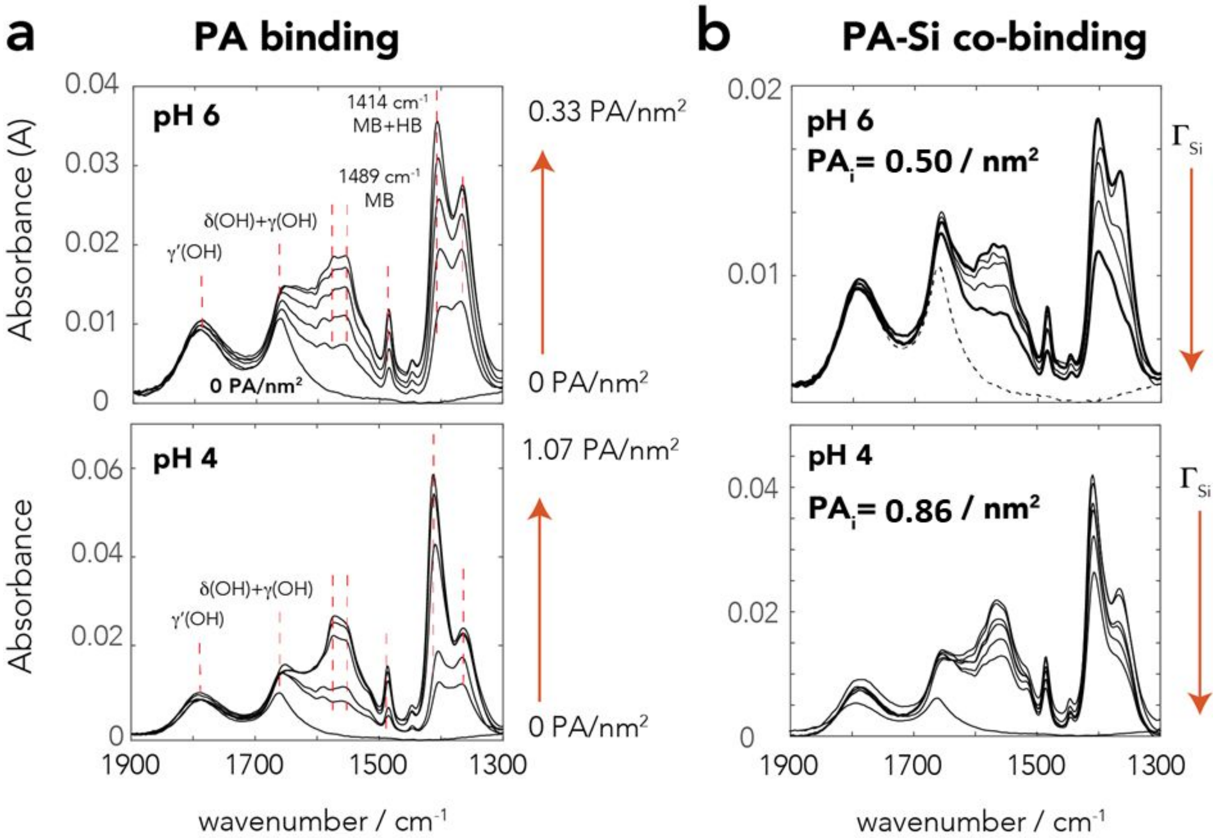

Figure S8. ATR-FTIR spectra of PA bound to N_2-dry (298 K) goethite, including overtones and combinations of goethite bulk bending modes (*cf.* Figure S2). Samples were initially equilibrated in 50 m^2/L suspensions in 10 mM NaCl at 298 K with(a) 0-400 μM PA and with (b)100 μM PA and 0-1000 μM Si at pH 4 and 6 for 1 d. Increased loadings are those of isotherms at these fixed pH values. We find symmetric (ν_{sym}=1369 cm^{-1}) and asymmetric (ν_{sym} = 1556 cm^{-1}) C-O stretching modes, C-OH (1404 cm^{-1}) stretching modes, C=C stretches ($\nu_{C=C}$ = 1597 cm^{-1}), and benzene ring vibrations (1489 and 1447 cm^{-1}).[9]

References

(1) Kanematsu, M.; Waychunas, G. A.; Boily, J.-F. Silicate Binding and Precipitation on Iron Oxyhydroxides. *Environ. Sci. Technol.* **2018**, *52* (4), 1827–1833. https://doi.org/10.1021/acs.est.7b04098.

(2) Hiemstra, T.; Barnett, M. O.; van Riemsdijk, W. H. Interaction of Silicic Acid with Goethite. *Journal of Colloid and Interface Science* **2007**, *310* (1), 8–17. https://doi.org/10.1016/j.jcis.2007.01.065.

(3) Song, X.; Boily, J.-F. Structural Controls on OH Site Availability and Reactivity at Iron Oxyhydroxide Particle Surfaces. *Physical Chemistry Chemical Physics* **2012**, *14* (8), 2579–2586. https://doi.org/10.1039/C2CP22715K.

(4) Song, X.; Boily, J.-F. Water Vapor Adsorption on Goethite. *Environ. Sci. Technol.* **2013**, *47* (13), 7171–7177. https://doi.org/10.1021/es400147a.

(5) Song, X.; Boily, J.-F. Water Vapor Interactions with FeOOH Particle Surfaces. *Chemical Physics Letters* **2013**, *560*, 1–9. https://doi.org/10.1016/j.cplett.2012.12.048.

(6) Boily, J.-F.; Szanyi, J.; Felmy, A. R. A Combined FTIR and TPD Study on the Bulk and Surface Dehydroxylation and Decarbonation of Synthetic Goethite. *Geochimica et Cosmochimica Acta* **2006**, *70* (14), 3613–3624. https://doi.org/10.1016/j.gca.2006.05.013.

(7) Ding, X.; Song, X.; Boily, J.-F. Identification of Fluoride and Phosphate Binding Sites at FeOOH Surfaces. *J. Phys. Chem. C* **2012**, *116* (41), 21939–21947. https://doi.org/10.1021/jp3083776.

(8) Boily, J.-F.; Felmy, A. R. On the Protonation of Oxo- and Hydroxo-Groups of the Goethite (α-FeOOH) Surface: A FTIR Spectroscopic Investigation of Surface O–H Stretching Vibrations. *Geochimica et Cosmochimica Acta* **2008**, *72* (14), 3338–3357. https://doi.org/10.1016/j.gca.2008.04.022.

(9) Hwang, Y. S.; Liu, J.; Lenhart, J. J.; Hadad, C. M. Surface Complexes of Phthalic Acid at the Hematite/Water Interface. *Journal of Colloid and Interface Science* **2007**, *307* (1), 124–134. https://doi.org/10.1016/j.jcis.2006.11.020.
